# Recent research on Novichok

**DOI:** 10.1007/s00204-022-03273-7

**Published:** 2022-03-10

**Authors:** Hermann M. Bolt, Jan G. Hengstler

**Affiliations:** Leibniz-Institut für Arbeitsforschung an der TU Dortmund IfADo, Ardeystr. 67, 44139 Dortmund, Germany

The first organophosphate intoxication cases that were published in Archives of Toxicology were caused by parathion (E605), and a high acute toxicity of this chemical was noted, at a human lethal dose of 2.1 g E605 forte (Vogel 1953). Since that, acetylcholine esterase inhibitors became a classical textbook matter in toxicology (reviews: Worek et al. 2016; Nepomivova and Kuca 2019).

Recently, the cases of Sergei and Yulia Skripal and of Alexei Navalny have attracted considerable public interest in acetylcholine esterase inhibitors in general, and Novichok agents in particular. Clinical details of both intoxication cases have been published, to which reference can be made (Skripal case: Vale et al. 2018; Navalny case: Steindl et al. 2021). Now, almost 2 and 4 years after the cases, it is interesting to discuss the available scientific research.

**Fig. 1:**
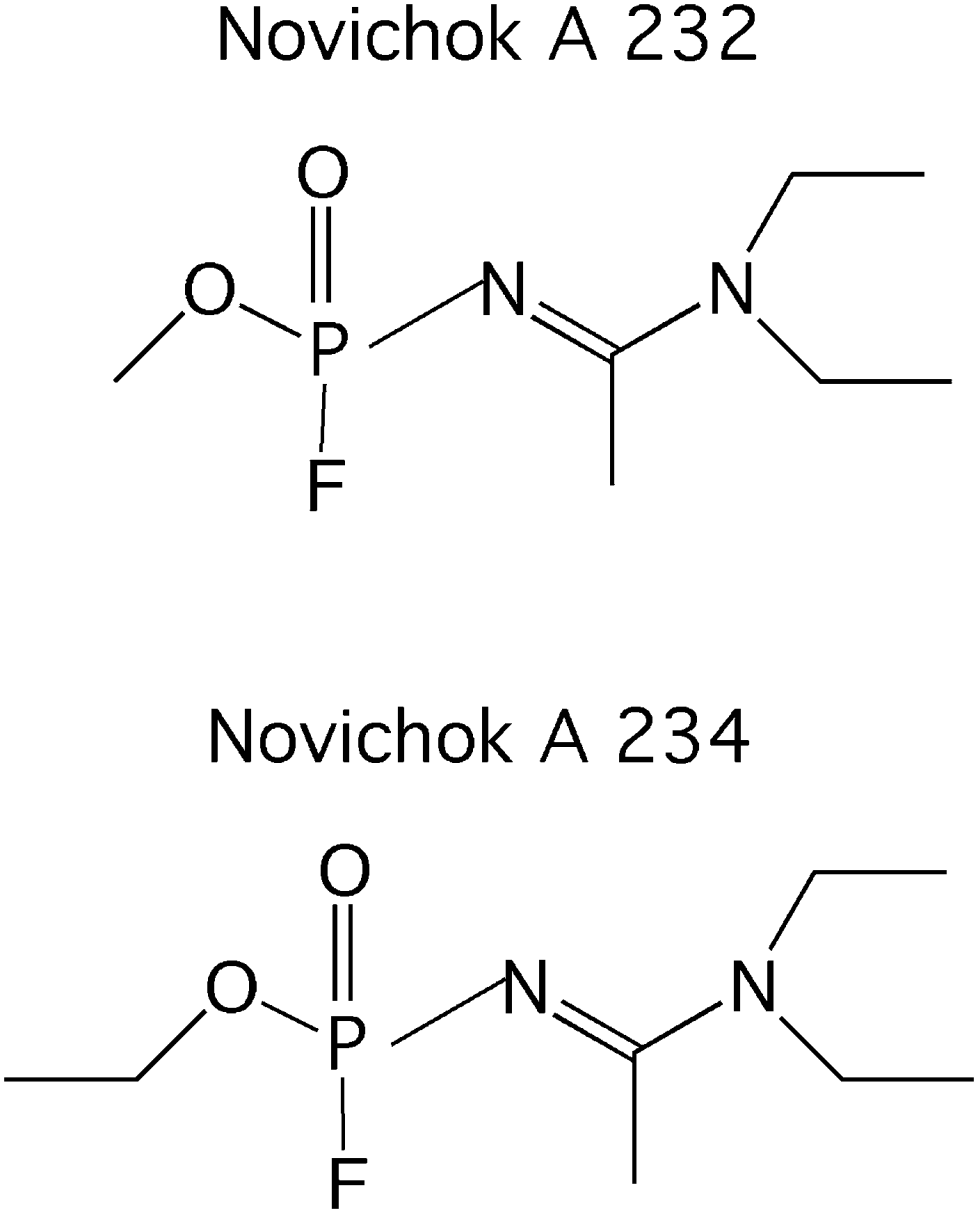
Structural formulae of the related Novichok agents A 232 and A 234 (Cai et al. 2018; Lee et al. 2021). A 234 has been identified as the agent of the Skripal case (Bhakhoa et al. 2019)

The Novichok agent A 234 (structural formula on Fig. 1) was alleged by the British government to have been used to poison the Skripals, and its identity was confirmed by the Organization for the Prohibition of Chemical Weapons (OPCW). After the British authorities classified this incident as poisoning terrorism based on the use of a novel series of nerve agents, OPCW ratified all Novichok-based components in the Chemical Weapons Convention lists in June 2020 (Lee et al. 2021).

In the Navalny case, the involvement of a Novichok agent and its biotransformation products was reported several days after establishing the diagnosis of cholinesterase inhibitor poisoning, when the patient had been transferred to Charité University Hospital in Berlin, Germany (Steindl et al. 2021).

The name “Novichok” is given to chemical warfare agents supposedly developed in the former Soviet Union between the 1970s and the 1990s, as a reaction to the British/American invention of VX agent. Designed to be undetectable and untreatable, these chemicals represent particularly toxic nerve agents and can, therefore, cause great harm if used as a weapon or if it falls into the hands of terrorists. Human exposure to Novichok agents is fatal unless aggressively managed. LD_50_ of Novichok agents is reported to be about 0.22 µg/kg b.w. (Franca et al. 2019; Chai et al. 2018).

At the time of the attack on the Skripals, information on Novichoks in the open literature was sparse. Chai et al. (2018) explained that “*most of what we understand of Novichok agents comes from testimony and memoirs of Dr. Vil S. Mirzayanov, the Chief of the Department of Counteraction against Foreign Technical Intelligence at the Russian State Union Scientific Research Institute for Organic Chemistry and Technology (GosNIIOKhT). Dr. Mirzayanov authored a 1994 report with the Stimson Center describing the state of chemical weapon disarmament in Russia. He detailed the initiation of a secret Soviet chemical weapons initiative to develop “newcomer” (i.e. Novichok in Russian) agents. The first three of these, Substance-33, A 230, and A 232, were produced at a GosNIIOKhT facility Russia using an organophosphate structural backbone. These three newcomer agents were synthesized much like VX, tabun, soman, and sarin, as unitary agents, meaning that the chemical structure is altered during production so that maximum potency occurs rapidly at the outset. Importantly, only binary agents (two inert substances that are combined prior to delivery to create the active nerve agent) that were effectively weaponized and tested were given the designator Novichok, while unitary agents and other organophosphates maintain their original designators… At the same time, the United States recognized the danger of stockpiling potent unitary agents, and initiated development of a Binary Internally Generated chemical weapon in the EYE series of canister weapons (BigEYE). In response, development of binary newcomer agents escalated at GosNIIOKhT, and in 1989 the first known binary newcomer agent, Novichok-5 was synthesized off the base structure of A-232*” (for detailed references, see Chai et al. 2018).

In fact, up to 2018 different structural formulae were proposed for key Novichok compounds in the open literature (Cai et al. 2018; Nepomivova and Kuca 2018). In the meantime, theoretical and mass spectrometry studies have appeared that ascertain the structures shown in Fig. 1 (Bhakkoa et al. 2019; Lee et al. 2021; Jeong et al. 2021).

Since 2018, in the toxicological literature, the following lines of investigation can be identified, which are reflected by key contributions to Archives of Toxicology.

(i) Clinical reports (Novichok) and improvement of therapy of intoxications by organophosphorus compounds (Hulse et al. 2019; Steindl et al. 2021; Vale et al. 2018; Worek et al. 2020; Zandona et al. 2020, 2021*):* a special focus is development of novel antidotes, e.g., novel oximes (Machamer et al 2020; Zandona et al. 2020, 2021) or engineered phoshotriesterases (Stigler et al. 2021). Given the world-wide incidents attributed to chemical weapons, such as Novichok agents, intensive-care clinicians should know how to rapidly recognize symptoms of acute poisoning and to administer life-saving antidotal therapy, when indicated (Chai et al. 2018).
(ii) Identification of modes of action of organophosphorus compounds on different organ systems: already Vogel (1953) has noted organophosphate effects on several organ systems. In the recent (January 2022) issue of Archives of Toxicology Tigges et al. (2022) used the observation that organophosphates may induce pneumonia and lung oedema as starting point to investigate underlying mechanisms. Rat precision-cut lung slices (PCLS) were exposed to parathion, malathion and their biotransformation products paraoxon and malaoxon (100–2000 µmol/L). Airway response, metabolic activity, release of LDH, cytokine expression and oxidative stress response were analyzed. A concentration-dependent inhibition of airway relaxation was observed after exposure with the oxon but not with the thion organophosphate. Cytotoxic effects were observed for both forms in higher concentrations. Increased cytokine expression was observed after exposure to parathion and paraoxon (IL-6, GM-CSF, and MIP-1α) and IL-6 expression was dependent on NFκB activation. Intracellular GSH levels were significantly reduced by all four tested organophosphates, but an increase in GSSG and HO-1 expression was predominantly observed after malaoxon exposure. Pretreatment with the antioxidant *N*-acetylcysteine reduced malaoxon but not paraoxon-induced cytotoxicity. PCLS as a 3D lung model system revealed organophosphate-induced effects depending on the particular organophosphate. The study may help to explain the variety of clinical outcomes induced by different organophosphates.
(iii) Application of computational/QSAR methods to study molecular aspects of Novichoks in particular and organophosphates in general (Bhakhoa et al. 2019; Jeong and Choi 2019; Harvey et al. 2020; Wang et al. 2021*):* as an example, Wang et al. (2021) applied quantitative structure–activity relationship (QSAR) models for predicting acute oral toxicity in rats and mice and inhibition constants concerning human acetylcholinesterase of 456 unique organophosphates. Based on robust, two-dimensional molecular descriptors and quantum chemical descriptors, which reflect electronic structures and reactivities, the influence of eight machine-learning algorithms on the prediction performance of QSAR models was explored, and consensus QSAR models were constructed. The consensus QSAR models exhibited robustness, practical prediction abilities, and wide application scopes. Poor correlation was observed between acute oral toxicity in mammals and the inhibition constants at the molecular level, indicating that the acute toxicity of organophosphates cannot be evaluated only by data of enzyme inhibitory activity; their toxicokinetic characteristics must also be considered. QSAR models may provide rapid theoretical assessment of the bioactivity of unstudied or unknown oganophosphates, as well as guidance for making regulatory decisions.
(iv) Analytical chemistry and biomonitoring: sophisticated analytical methods, such as LC–MS/MS, must be used to analytically characterize Novichok agents (e.g., A 234). Recent progress in this direction has been reported (Lee et al. 2021). Another promising avenue of research is blood protein adductomics (Sabbioni and Day 2022). Here, reference can be made to a recent review by Golime et al. (2019). These authors expect that the adductomics approach is useful to support the characterization of the adducts of unknown chemical warfare agents. Rapid advances in mass spectrometry to acquire high-quality data with greater resolution will enable analysis of protein and DNA adducts of xenobiotics including chemical warfare agents. In this context, Jeong et al. (2021) investigated nonapeptide adducts of Novichok A 232 an A 234 to the active serine site of butyrylcholine esterase, which plays a key role in enzyme activity, using LC–MS/HRMS. Biomarkers exhibited a significant amount of fragment ions from the inhibiting agent. These biomarkers had a higher abundance of [M + 2H]^2+^ ions than [M + H]^+^ ions, making doubly charged ions suitable for trace analysis. Thus, adductomics may serve as a powerful bioanalytical tool for the verification of exposure to chemical warfare agents in the future.

Archives of Toxicology will continue to publish new and relevant data along such research lines.
